# Novel proteins associated with risk for coronary heart disease or stroke among postmenopausal women identified by in-depth plasma proteome profiling

**DOI:** 10.1186/gm169

**Published:** 2010-07-28

**Authors:** Ross L Prentice, Sophie Paczesny, Aaron Aragaki, Lynn M Amon, Lin Chen, Sharon J Pitteri, Martin McIntosh, Pei Wang, Tina Buson Busald, Judith Hsia, Rebecca D Jackson, Jacques E Rossouw, JoAnn E Manson, Karen Johnson, Charles Eaton, Samir M Hanash

**Affiliations:** 1 Division of Public Health Sciences, Fred Hutchinson Cancer Research Center, 1100 Fairview Ave N., Seattle, WA 98102, USA; 2 Department of Pediatrics, University of Michigan Comprehensive Cancer Center, 1500 East Medical Center Drive, Ann Arbor, MI 48109, USA; 3 Research and Development, AstraZeneca LP, 1971 Rockland Road, Wilmington, DE 19803, USA; 4 Division of Endocrinology, Ohio State University, 198 McCampbell, 1581 Dodd Drive, Columbus, OH 43210, USA; 5 WHI Project Office, National Heart, Lung, and Blood Institute, National Institutes of Health, 6701 Rockledge Drive, Bethesda, MD 20892, USA; 6 Division of Preventive Medicine, Brigham and Women's Hospital, Harvard Medical School, 75 Francis Street, Boston, MA 02115, USA; 7 Department of Preventive Medicine, University of Tennessee Health Sciences Center, 66 N. Pauline, Memphis, TN 38163, USA; 8 Brown University, Memorial Hospital of Rhode Island, 111 Brewster Street, Pawtucket, RI 02860, USA

## Abstract

**Background:**

Coronary heart disease (CHD) and stroke were key outcomes in the Women's Health Initiative (WHI) randomized trials of postmenopausal estrogen and estrogen plus progestin therapy. We recently reported a large number of changes in blood protein concentrations in the first year following randomization in these trials using an in-depth quantitative proteomics approach. However, even though many affected proteins are in pathways relevant to the observed clinical effects, the relationships of these proteins to CHD and stroke risk among postmenopausal women remains substantially unknown.

**Methods:**

The same in-depth proteomics platform was applied to plasma samples, obtained at enrollment in the WHI Observational Study, from 800 women who developed CHD and 800 women who developed stroke during cohort follow-up, and from 1-1 matched controls. A plasma pooling strategy, followed by extensive fractionation prior to mass spectrometry, was used to identify proteins related to disease incidence, and the overlap of these proteins with those affected by hormone therapy was examined. Replication studies, using enzyme-linked-immunosorbent assay (ELISA), were carried out in the WHI hormone therapy trial cohorts.

**Results:**

Case versus control concentration differences were suggested for 37 proteins (nominal *P *< 0.05) for CHD, with three proteins, beta-2 microglobulin (B2M), alpha-1-acid glycoprotein 1 (ORM1), and insulin-like growth factor binding protein acid labile subunit (IGFALS) having a false discovery rate < 0.05. Corresponding numbers for stroke were 47 proteins with nominal *P *< 0.05, three of which, apolipoprotein A-II precursor (APOA2), peptidyl-prolyl isomerase A (PPIA), and insulin-like growth factor binding protein 4 (IGFBP4), have a false discovery rate < 0.05. Other proteins involved in insulin-like growth factor signaling were also highly ranked. The associations of B2M with CHD (*P *< 0.001) and IGFBP4 with stroke (*P *= 0.005) were confirmed using ELISA in replication studies, and changes in these proteins following the initiation of hormone therapy use were shown to have potential to help explain hormone therapy effects on those diseases.

**Conclusions:**

In-depth proteomic discovery analysis of prediagnostic plasma samples identified B2M and IGFBP4 as risk markers for CHD and stroke respectively, and provided a number of candidate markers of disease risk and candidate mediators of hormone therapy effects on CHD and stroke.

**Clinical Trials Registration:**

ClinicalTrials.gov identifier: NCT00000611

## Background

Blood protein concentrations provide a source for novel disease risk markers that may be modifiable by treatments or other exposures. As such, protein markers have potential to enhance the understanding of disease pathogenesis, and to elucidate biological processes whereby an exposure affects disease risk.

We report here on a large-scale proteomic study that aimed to uncover novel associations between plasma proteins and the risk of subsequent coronary heart disease (CHD) or stroke. These diseases were key outcomes in Women's Health Initiative (WHI) randomized postmenopausal hormone therapy trials of 0.625 mg/d conjugated equine estrogen (E-alone), or this same preparation plus 2.5 mg/d medroxyprogesterone acetate (E+P). We also sought to identify proteins that both distinguished cases from controls and were altered by E-alone or E+P as candidate biomarkers for elucidation of hormone therapy effects on these diseases [1-6]. E-alone and E+P were each found to yield an elevation in stroke risk [3,4], whereas E+P effects were unfavorable, and unfavorable compared to E-alone effects, for CHD [5,6]. A related research effort is considering case versus control comparisons for breast cancer [7,8].

We recently reported blood proteomic changes between baseline and 1 year for 50 women assigned to active treatment in each of the E-alone and E+P trials [9,10]. An intact protein analysis system (IPAS) [11-14] was used for these analyses. Under stringent criteria for protein identification and relative quantification, 378 proteins were quantified [10]. There was some evidence (nominal *P *< 0.05) of change from baseline to 1 year with either or both of E-alone and E+P for a remarkable 44.7% of these proteins. These proteins were involved in coagulation, inflammation, immune response, metabolism, cell adhesion, growth factors, and osteogenesis; pathways that plausibly relate to observed clinical effects [1-8] for these regimens.

A comparatively larger number of study subjects is needed to detect modest associations between plasma proteins and subsequent risk of CHD or stroke. Hence, we contrasted pools formed by equal plasma volumes from 100 cases or from 100 pair-matched controls, with eight such pool pairs for each of the study diseases. We report here on proteins, and sets of proteins, having evidence of a case-control difference in plasma concentration for CHD or stroke, and on the overlap of these proteins with those altered by E-alone or E+P. Enzyme-linked-immunosorbent assay (ELISA) replication studies in the WHI hormone therapy trial cohorts were carried out subsequently for selected proteins.

## Methods

### Study subjects and outcome ascertainment

Cases and controls were drawn from the WHI observational study, a prospective cohort study of 93,676 postmenopausal women in the age range 50 to 79 years at enrollment during 1993 to 1998 [15,16]. Fasting blood specimens were obtained at baseline as a part of eligibility screening. Serum and plasma samples were shipped to a central repository and stored at -70°C. Disease events during cohort follow-up were initially self-reported, followed by physician adjudication at participating WHI clinical centers, and central adjudication of some outcomes [17]. CHD was composed of myocardial infarction and death due to coronary disease. Cases of hospitalized stroke were based on rapid neurologic deficit attributable to obstruction or rupture of the arterial system or on a demonstrable lesion compatible with acute stroke. CHD and stroke cases were chosen as the earliest 800 incident cases during cohort follow-up for which a suitable plasma specimen was available. Each case was 1-1 matched to a control woman who did not develop any of the study diseases during cohort follow-up. Cases and controls were matched on baseline age (within 1 year), self-reported ethnicity, hysterectomy status, prior history of the study disease, and enrollment date (median difference 1 month). Non-overlapping sets of controls were chosen for CHD, stroke, and breast cancer. Diagnosis occurred an average of 2.2 and 4.5 years after blood draw for the CHD and stroke cases, respectively.

### Sample preparation, protein fractionation, and mass spectrometry analysis

We used 3,200 patient samples (800 stroke cases, 800 CHD cases, and 1,600 controls) to form case and control pool pairs for 16 IPAS experiments (8 stroke + 8 CHD). For each IPAS experiment, a case and control pool was created using 5 μl of EDTA plasma for each of the 100 cases or 100 controls for proteomic analysis. The pools were independent, with each sample used in only one pool. The IPAS analytic methods used for this project have been described [13] and detailed information is available in Additional file 1. Following immunodepletion of the six most abundant proteins (albumin, IgG, IgA, transferrin, haptoglobin, antitrypsin), pools were concentrated and case and control pools were isotopically labeled with either the 'light' C12 or the 'heavy' C13 acrylamide. The case and corresponding control pools were then mixed together for further analysis.

The combined sample was diluted, and each sample was separated into eight fractions using anion exchange chromatography, and each fraction was further separated using reversed-phase chromatography.

Lyophilized aliquots from the reversed-phase fractionation were subjected to in-solution trypsin digestion, and individual digested fractions from each reversed-phase run were combined, giving a total of 96 (8 × 12 reversed-phase) fractions for analysis from each original mixed case and control pool. Tryptic peptides were analyzed by a LTQ-FT mass spectrometer. Spectra were acquired in a data-dependent mode in a mass/charge range of 400 to 1,800, and the 5 most abundant + 2 or + 3 ions were selected from each spectrum for tandem mass spectrometry (MS/MS) analysis.

### Protein identification and case versus control concentration assessment

The acquired liquid chromatography MS/MS data were processed by a Computational Proteomics Analysis System [18]. Database searches were performed using X!Tandem against the human International Protein Index (IPI) using tryptic search [18]. Database search results were analyzed using PeptideProphet [19] and ProteinProphet [20]. Protein identification was based on ProteinProphet scores that indicate an error rate of less than 10%.

The relative quantification of case versus control concentration for cysteine-containing peptides (acrylamide label binds to cysteine) identified by MS/MS was extracted using a script [11] that calculates the relative peak areas of heavy to light acrylamide-labeled peptides; see [10] for further details. Proteins from all IPAS experiments for a specific disease were aligned by their protein group number, assigned by ProteinProphet, in order to identify master groups of indistinguishable proteins across experiments. Ratios for these protein groups were logarithmically transformed and median-centered at zero for each IPAS experiment. Groups that had fewer than four peptide ratios across all experiments for a specific disease, groups that contained proteins that were targeted for depletion, and groups in which all proteins had been annotated as 'defunct' by IPI, were excluded from analysis.

### Statistical analysis of case versus control protein concentrations

Data analysis was based on log(base2) concentration ratios from case versus control pools. The log-ratios for a particular protein were analyzed using linear models that included a disease-specific mean parameter plus a variable defined as 1 if the heavy acrylamide label was assigned to the case group and -1 otherwise. A weighted moderated *t*-test [21], implemented in the R package LIMMA [22], was used to examine whether there was evidence of a disease-specific mean parameter that differs from zero, after adjusting for any labeling effect. The log-ratios were weighted by the number of quantified peptides for each protein. Log-ratios for all three diseases were used to jointly estimate model parameters (the heavy acrylamide label was randomly assigned to the case or control pool for both stroke and breast cancer, and to the case pools for CHD), and to increase the degrees of freedom for log-ratio variance estimation. One of the breast cancer pool pairs gave log-ratios that were comparatively highly variable, and is excluded from all analyses. Benjamini and Hochberg's method [23] was used to accommodate multiple testing, through the calculation of estimated false discovery rates (FDRs), separately for each study disease.

### Biological pathway analyses

A regularized Hotelling T^2 ^procedure was used to identify sets of proteins, defined by biological pathways, that differ in concentrations between cases and controls for each study disease. This testing procedure takes advantage of the correlation structure among the log-ratios for proteins in a given set. Protein sets were defined using the Kyoto Encyclopedia of Genes and Genomes (KEGG) database [24,25].

### ELISA replication analyses

Selected protein associations with disease risk were further evaluated by ELISA testing of CHD and stroke cases and controls drawn from the non-overlapping WHI hormone therapy trial cohorts. Baseline plasma samples were evaluated for women who developed CHD or stroke during the first year following randomization, along with 1-1 matched disease-free controls. Matching variables included age, randomization date, hysterectomy status, and prevalent study disease. Assays were performed according to manufacturer's direction, for beta-2 microglobulin (B2M; Genway San Diego, CA, USA) and insulin-like growth factor binding protein 4 (IGFBP4; R & D Systems Minneapolis, MN, USA). All samples were assayed with sample characteristics blinded and in duplicate.

## Results

### Plasma protein risk markers

Additional file 2 provides information on baseline characteristics for the 800 CHD and 800 stroke cases and their non-overlapping 1-1 matched controls. All women were postmenopausal and in the age range 50 to 79 years at recruitment. Most were white. About two-thirds were overweight or obese. There were few current cigarette smokers. Sixteen percent of CHD cases had experienced a myocardial infarction and 15% of stroke cases had experienced a stroke prior to WHI enrollment.

Case versus control concentration ratios were determined following application of stringent standards for identification and quantification (see Methods). Following application of an additional requirement that proteins were quantified for at least two of the pool pairs for a disease, 346 proteins for CHD and 366 proteins for stroke were included in statistical analyses. Of these, a total of 37 proteins have nominal significance levels of *P *< 0.05 for CHD cases versus controls, compared to 17.3 expected by chance; and 47 have *P *< 0.05 for stroke cases versus controls, compared to 18.3 expected by chance. These proteins are listed in Tables 1 and 2 along with their mean log-intensity ratios, *P*-values, and FDRs.

**Table 1:** Proteins having some evidence (*P *< 0.05) of difference in concentration between coronary heart disease cases and controls

Protein	Description	Log(base2) case vs control ratio	***P*-value**^**a**^	**FDR**^**a**^
B2M	Beta-2-microglobulin.	0.212	5.07e-05	0.0176
ORM1	Alpha-1-acid glycoprotein 1	0.120	0.000182	0.0315
IGFALS	Insulin-like growth factor-binding protein complex acid labile chain	-0.112	0.000384	0.0443
THBS1	Thrombospondin-1	-0.632	0.00133	0.0749
LPA	Apolipoprotein(A)	0.347	0.00138	0.0749
CFD	Complement factor D preproprotein	0.210	0.00141	0.0749
PRG4	Isoform C of proteoglycan 4	0.232	0.00152	0.0749
GPX3	Glutathione peroxidase 3	-0.224	0.00308	0.133
IGFBP1	Insulin-like growth factor-binding protein 1	0.423	0.00381	0.146
MST1	Hepatocyte growth factor-like protein homolog	-0.306	0.00592	0.205
ITIH2	Inter-alpha-trypsin inhibitor heavy chain H2	-0.140	0.00786	0.247
ENO1	Isoform alpha-enolase of alpha-enolase	-0.418	0.00950	0.255
C9	Complement component C9	0.0827	0.00989	0.255
SFTPB	Pulmonary surfactant-associated protein B precursor	0.551	0.0112	0.255
FHL1	cDNA FLJ55259 highly similar to four and a half lim domains protein 1	-0.481	0.0116	0.255
CRISP3	cDNA FLJ75207	0.147	0.0118	0.255
SERPIND1	Serpin peptidase inhibitor clade D (heparin cofactor) member 1	0.210	0.0176	0.334
CD5L	CD5 antigen-like	0.152	0.0181	0.334
SOD3	Extracellular superoxide dismutase [Cu-Zn]	0.453	0.0183	0.334
TPI1	Triosephosphate isomerase 1 isoform 2	-0.144	0.0232	0.401
C1QB	Complement component 1 Q subcomponent B chain precursor	-0.106	0.0271	0.407
ATRN	Isoform 1 of attractin	-0.151	0.0274	0.407
INHBE	Inhibin beta E chain	0.384	0.0284	0.407
CHRDL2	Isoform 2 of chordin-like protein 2	-0.647	0.0287	0.407
LIMS1	cDNA FLJ55516 highly similar to particularly interesting new Cys-His protein	-0.412	0.0318	0.407
VASP	Vasodilator-stimulated phosphoprotein	-0.499	0.0356	0.407
C8A	Complement component C8 alpha chain	0.170	0.0359	0.407
C2	Complement C2 (fragment)	-0.230	0.0361	0.407
CD14	Monocyte differentiation antigen CD14	0.105	0.0361	0.407
GC	Vitamin D-binding protein	-0.0451	0.0364	0.407
MTPN	Myotrophin	-0.240	0.0372	0.407
SERPINF2	Serpin peptidase inhibitor, clade F, member 2	-0.110	0.0383	0.407
ACTA2	Actin aortic smooth muscle	-1.22	0.0388	0.407
TAGLN2	Transgelin-2	-0.186	0.0426	0.433
FERMT3	Isoform 2 of fermitin family homolog 3	-0.560	0.0462	0.454
F12	Coagulation factor XII	-0.147	0.0472	0.454
AFM	Afamin	-0.0764	0.0490	0.458

^a^*P*-value = significance level for no difference in protein concentration; FDR = estimated false discovery rate.

**Table 2:** Proteins having some evidence (*P *< 0.05) of difference in concentration between stroke cases and controls

Protein	Description	Log(base2) case vs control ratio	***P*-value**^**a**^	**FDR**^**a**^
APOA2	Apolipoprotein A-II	-0.120	2.71e-05	0.00991
PPIA	Peptidyl-prolyl cis-trans isomerase A	0.194	7.68e-05	0.0141
IGFBP4	Insulin-like growth factor-binding protein 4	0.409	0.000320	0.0391
F2	Prothrombin (fragment)	-0.0732	0.000702	0.0642
IGF2	Isoform 1 of insulin-like growth factor II	-0.0694	0.00225	0.138
C6	Complement component 6 precursor	-0.140	0.00227	0.138
LILRA3	Leukocyte immunoglobulin-like receptor subfamily a member 3	0.316	0.00341	0.177
HPX	Hemopexin	-0.0448	0.00407	0.177
IGFBP6	Insulin-like growth factor-binding protein 6	0.667	0.00435	0.177
LOC650157	Similar to peptidyl-pro cis trans isomerase	0.237	0.00510	0.187
IGFBP2	Insulin-like growth factor-binding protein 2	0.480	0.00609	0.189
GC	Vitamin D-binding protein	-0.0532	0.00699	0.189
CADM1	Isoform 1 of cell adhesion molecule 1	-0.199	0.00762	0.189
PIN1	Peptidyl-prolyl cis-trans isomerase NIMA-interacting 1	0.190	0.00767	0.189
CTSD	Cathepsin D	0.490	0.00776	0.189
COL1A1	Collagen alpha-1(I) chain	0.195	0.00826	0.189
F13B	Coagulation factor XIII b chain	0.121	0.00903	0.194
MANSC1	MANSC domain-containing protein 1	-0.962	0.0102	0.207
COL6A3	Isoform 1 of collagen alpha-3(VI) chain	0.828	0.0109	0.210
GRN	cDNA FLJ13286 fis clone OVARC1001154 highly similar to *Homo sapiens *clone 24720 epithelin 1 and 2 mRNA	0.316	0.0130	0.238
RNASE1	Ribonuclease pancreatic	0.582	0.0143	0.243
MTPN	Myotrophin	0.249	0.0146	0.243
GLIPR2	Golgi-associated plant pathogenesis-related protein 1	0.623	0.0168	0.265
ADAMTSL2	ADAMTS-like protein 2	0.205	0.0184	0.265
ITIH4	Isoform 2 of inter-alpha-trypsin inhibitor heavy chain H4	-0.238	0.0187	0.265
HLA-DRB5^b^	Non-secretory ribonuclease	0.784	0.0188	0.265
KLKB1	Plasma kallikrein	-0.115	0.0202	0.270
CD59	CD59 glycoprotein	0.866	0.0208	0.270
CD14	Monocyte differentiation antigen CD14	0.104	0.0214	0.270
CSF1R	Macrophage colony-stimulating factor 1 receptor	0.259	0.0223	0.272
GRB2	Isoform 1 of growth factor receptor-bound protein 2	1.58	0.0235	0.278
CD5L	CD5 antigen-like	0.147	0.0253	0.289
B2M	Beta-2-microglobulin	0.0728	0.0280	0.310
SERPINC1	Antithrombin-III	-0.0631	0.0312	0.325
FCN3	Isoform 1 of ficolin-3	0.132	0.0323	0.325
HGFAC	Hepatocyte growth factor activator	-0.592	0.0324	0.325
RBP4	Retinol-binding protein 4	0.0478	0.0346	0.325
CFHR5	Complement factor H-related 5	-0.0800	0.0348	0.325
PRDX2	Peroxiredoxin-2	-0.533	0.0361	0.325
C8A	Complement component C8 alpha chain	-0.179	0.0373	0.325
ADAMTSL4	Isoform 1 of ADAMTS-like protein 4	-0.130	0.0373	0.325
QSOX1	Isoform 1 of sulfhydryl oxidase 1	0.370	0.0376	0.325
CPB2	Isoform 1 of carboxypeptidase B2	-0.228	0.0381	0.325
FETUB	Fetuin-B	0.0662	0.0410	0.332
PPIF	Peptidyl-prolyl cis-trans isomerase mitochondrial	0.318	0.0414	0.332
LCN2	Neutrophil gelatinase-associated lipocalin	0.172	0.0417	0.332
DSC1	Isoform 1B of desmocollin-1	-0.265	0.0438	0.341

^a^*P*-value = significance level for no difference in protein concentration; FDR = estimated false discovery rate. ^b^The DRB5 protein group also includes ZNF749, LOC100133811, LOC100133484, LOC100133661, HLA-DRB1, HLA-DRB4, RNASE2, and HLA-DRB3.

**Figure 1:**
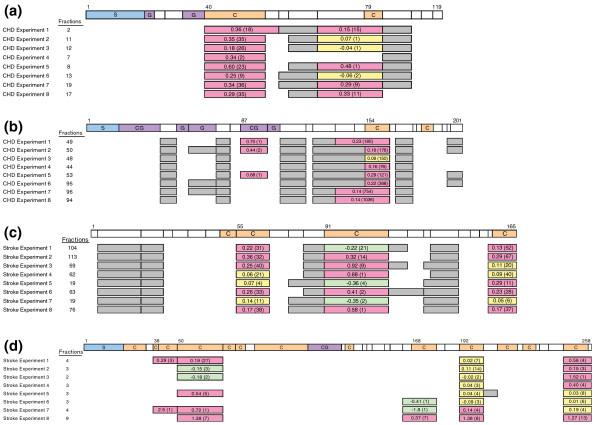
**Identification and quantitative analysis of peptides in plasma**. From CHD cases and controls in eight experiments for **(a) **beta-2 microglobulin (B2M) and **(b) **alpha-1-acid glycoprotein 1 (ORM1); and from stroke cases and controls in eight experiments for **(c) **peptidyl-prolyl isomerase A (PPIA) and **(d) **insulin-like growth factor binding protein 4 (IGFBP4). Tryptic peptides from the amino terminus (1) to the carboxyl terminus are shown at the top. S, C and G indicate signal peptide, cysteine-containing and glycosylated peptides, respectively. Peptides identified, but which lack cysteine for quantification, are shown in gray. The log2 case/control ratio is shown for cysteine-containing peptides with the number of MS events for that peptide shown in parentheses. The number of plasma fractions where each peptide was quantified is indicated.

Proteins having small FDRs are likely to be associated with disease risk. Three proteins, B2M, alpha-1-acid glycoprotein 1 (ORM1), and insulin-like growth factor binding protein, acid labile subunit (IGFALS) have a FDR < 0.05 for association with CHD risk; and three proteins, apolipoprotein A-II precursor (APOA2), peptidyl-prolyl isomerase A (PPIA), and IGFBP4 have a FDR < 0.05 for association with stroke risk. Six other proteins have a FDR < 0.20 for CHD association, and 14 have a FDR < 0.20 for stroke association. Figure 1 shows peptide coverage and case versus control concentration ratios for B2M, ORM1, PPIA, and IGFBP4 separately for each plasma pool pair. Additional files 3 and 4 show *P*-values and FDRs for the entire set of proteins quantified separately for the CHD and stroke analyses. These tables also provide information on the number of peptides and unique peptides identified, and on the number of peptides and unique peptides quantified for each listed protein. IPI numbers corresponding to the gene/protein are also listed.

### Protein levels that are also affected by postmenopausal hormone therapy

Table 3 shows the subset of Table 1 proteins that appeared to have concentrations affected (*P *< 0.05) by one or both of E+P or E-alone in earlier proteomic discovery work [10], while Table 4 provides this information for the corresponding subset of Table 2. Five of the 6 proteins having a FDR < 0.05 for disease association are influenced by hormone therapy. In addition to these, certain other IGF binding proteins are evidently influenced by hormone therapy and may be related to CHD (IGFBP1) or stroke (IGFBP2, IGFBP6).

**Table 3:** Proteins having some evidence (*P *< 0.05) of concentration difference between CHD cases and controls that are altered (*P *< 0.05) by postmenopausal hormone therapy

		CHD			E+P		E-alone	
				
Protein	Description	Log(base2) case vs control ratio	***P*-value**^**a**^	**FDR**^**a**^	Log(base2) case vs control ratio	***P*-value**^**a**^	Log(base2) case vs control ratio	***P*-value**^**a**^
B2M	Beta-2-microglobulin	0.212	5.07e-05	0.0176	0.208	0.00205	0.230	0.00110
IGFALS	Insulin-like growth factor-binding protein complex acid labile chain	-0.112	0.000384	0.0443	0.151	0.00785	0.143	0.0282
CFD	Complement factor D preproprotein	0.210	0.00141	0.0749	-0.246	0.00871	-0.0472	0.620
PRG4	Isoform C of proteoglycan 4	0.232	0.00152	0.0749	0.0735	0.181	0.128	0.0327
IGFBP1	Insulin-like growth factor-binding protein 1	0.423	0.00381	0.146	0.528	0.00242	1.270	3.66e-06
MST1	Hepatocyte growth factor-like protein homolog	-0.306	0.00592	0.205	0.530	0.0100	0.633	0.00195
C9	Complement component C9	0.0827	0.00989	0.255	0.101	0.0645	0.179	0.00858
SERPIND1	Serpin peptidase inhibitor clade D (heparin cofactor) member 1	0.210	0.0176	0.334	0.450	0.0240	0.156	0.344
C1QB	Complement component 1 Q subcomponent B chain precursor	-0.106	0.0271	0.407	0.0113	0.465	0.0480	0.0125
ATRN	Isoform 1 of attractin	-0.151	0.0274	0.407	-0.190	0.000213	-0.126	0.00366
INHBE	Inhibin beta E chain	0.384	0.0284	0.407	0.258	0.0723	0.520	0.00734
CHRDL2	Isoform 2 of chordin-like protein 2	-0.647	0.0287	0.407	-0.301	0.0415	-0.000906	0.993
C8A	Complement component C8 alpha chain	0.170	0.0359	0.407	-0.206	0.000163	-0.202	0.000121
C2	Complement C2 (fragment)	-0.230	0.0361	0.407	0.334	0.00371	0.291	0.0107
GC	Vitamin D-binding protein	-0.0451	0.0364	0.407	0.231	3.10e-06	0.237	2.75e-06
SERPINF2	Serpin peptidase inhibitor, clade F, member 2	-0.110	0.0383	0.407	0.0922	0.148	0.166	0.0247
F12	Coagulation factor XII	-0.147	0.0472	0.454	0.261	0.000102	0.252	0.000219
AFM	Afamin	-0.0764	0.0490	0.458	0.0580	0.119	0.177	0.000330

^a^*P*-value = significance level for no difference in protein concentration; FDR = estimated false discovery rate.

**Table 4:** Proteins having some evidence (*P *< 0.05) of concentration difference between stroke cases and controls that are altered (*P *< 0.05) by postmenopausal hormone therapy

		Stroke			E+P		E-alone	
				
Protein	Description	Log(base2) case vs control ratio	***P*-value**^**a**^	**FDR**^**a**^	Log(base2) case vs control ratio	***P*-value**^**a**^	Log(base2) case vs control ratio	***P*-value**^**a**^
APOA2	Apolipoprotein A-II	-0.120	2.71e-05	0.00991	0.212	0.000532	0.302	1.75e-05
PPIA	Peptidyl-prolyl cis-trans isomerase A	0.194	7.68e-05	0.0141	0.381	0.00899	0.201	0.126
IGFBP4	Insulin-like growth factor-binding protein 4	0.409	0.000320	0.0391	0.179	0.102	0.511	0.000697
F2	Prothrombin (fragment)	-0.0732	0.000702	0.0642	0.0633	0.00366	0.0282	0.138
C6	Complement component 6 precursor	-0.140	0.00227	0.138	-0.123	0.00151	-0.171	0.000123
LILRA3	Leukocyte immunoglobulin-like receptor subfamily A member 3	0.316	0.00341	0.177	-0.237	0.00874	-0.281	0.000277
HPX	Hemopexin	-0.0448	0.00407	0.177	0.123	6.65e-05	0.117	0.000124
IGFBP6	Insulin-like growth factor-binding protein 6	0.667	0.00435	0.177	0.0868	0.235	0.207	0.0158
IGFBP2	Insulin-like growth factor-binding protein 2	0.480	0.00609	0.189	-0.420	0.00477	-0.287	0.0317
GC	Vitamin D-binding protein	-0.0532	0.00699	0.189	0.231	3.10e-06	0.237	2.75e-06
CADM1	Isoform 1 of cell adhesion molecule 1	-0.199	0.00762	0.189	-0.0139	0.875	0.180	0.0249
COL1A1	Collagen alpha-1(I) chain	0.195	0.00826	0.189	-0.896	5.40e-07	-0.575	8.80e-05
COL6A3	Isoform 1 of collagen alpha-3(VI) chain	0.828	0.0109	0.210	-0.197	0.00852	-0.0134	0.834
RNASE1	Ribonuclease pancreatic	0.582	0.0143	0.243	0.0346	0.311	0.0953	0.0427
ITIH4	Isoform 2 of inter-alpha-trypsin inhibitor heavy chain H4	-0.238	0.0187	0.265	0.458	0.000733	0.374	0.00495
KLKB1	Plasma kallikrein	-0.115	0.0202	0.270	0.252	0.00208	0.230	0.00187
B2M	Beta-2-microglobulin	0.0728	0.0280	0.310	0.208	0.00205	0.230	0.00110
SERPINC1	Antithrombin-III	-0.0631	0.0312	0.325	-0.196	5.05e-06	-0.143	5.50e-05
FCN3	Isoform 1 of ficolin-3	0.132	0.0323	0.325	0.0351	0.0287	0.0357	0.0333
HGFAC	Hepatocyte growth factor activator	-0.592	0.0324	0.325	-0.191	0.0979	-0.308	0.00765
RBP4	Retinol-binding protein 4	0.0478	0.0346	0.325	0.167	0.000117	0.177	0.000262
CFHR5	Complement factor H-related 5	-0.0800	0.0348	0.325	0.179	0.000264	0.241	2.76e-05
PRDX2	Peroxiredoxin-2	-0.533	0.0361	0.325	0.691	0.0201	-0.0266	0.925
C8A	Complement component C8 alpha chain	-0.179	0.0373	0.325	-0.206	0.000163	-0.202	0.000121
FETUB	Fetuin-B	0.0662	0.0410	0.332	0.783	1.09e-09	0.741	1.02e-09

^a^*P*-value = significance level for no difference in protein concentration; FDR = estimated false discovery rate.

### Protein set (pathway) analyses

For each disease, we focused attention on KEGG pathways for which relative quantification was available for three or more proteins and tested for evidence of a case versus control difference in plasma concentrations for the set of quantified proteins. For CHD there were two pathways having *P *< 0.05, namely a mitogen-activated protein kinase (MAPK) signaling pathway (*P *= 0.02), which included six quantified proteins (NTRK2, FLNA, CD14, TGFB1, FGFR1, and CACNA2D1), and a glycolysis and gluconeogenesis metabolic pathway (*P *= 0.03), which included nine quantified proteins (LDHB, LDHA, PKM2, ALDOA, ALDOC, TPI1, GAPDH, ENO1, PGK1). The FDRs were 0.09 for both pathways.

**Figure 2:**
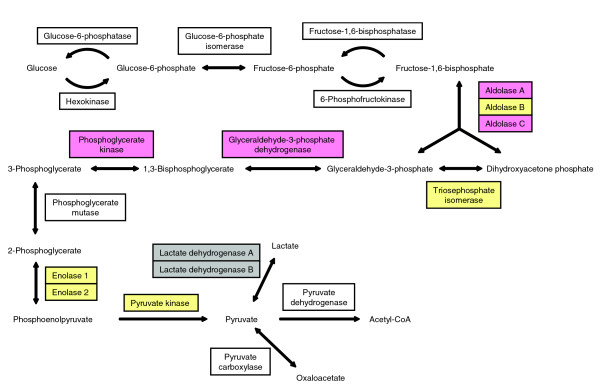
**Glycolysis/gluconeogenesis pathway**. Enzymes identified in stroke experiments are indicated by shading. Red and yellow indicate increased and no change in cases compared to controls, respectively. Gray indicates proteins identified but not quantified.

In comparison, there were six pathways having *P *< 0.05 for stroke; four of which had a FDR < 0.05. These four were a hematopoietic cell lineage pathway (CD44, GP1BA, C5F1R, CD59, CD14), a purine metabolism pathway (AK1, AK2, PKM2), a peroxisome proliferator-activated receptor signaling pathway (APOA2, FABP4, FABP1), and a glycolysis and gluconeogenesis pathway having a set of quantified proteins (PKM2, ALDOA, ALDOC, ALDOB, TPI1, ENO2, GAPDH, ENO1, PGK1) that strongly overlaps that listed above for CHD. Figure 2 shows the substantial peptide coverage of glycolytic pathway proteins in the stroke IPAS experiments.

### ELISA replication studies

B2M is of specific interest for CHD in view of higher levels in cases versus controls, and higher levels following 1-year of use of either E+P or E-alone (Table 3). IGFBP4 is of specific interest for stroke for these same reasons (Table 4). Hence, these proteins were selected for ELISA replication studies in the WHI hormone therapy trial cohorts.

Based on individual plasma samples from 106 CHD cases occurring during the first year following randomization in the hormone therapy trials, and from 1-1 matched controls, ELISA evaluation yielded B2M concentrations that were 17.9% higher (*P *< 0.001) in cases versus controls (geometric mean of log-ratios of 1.179 with 95% confidence interval (CI) of 1.107 to 1.290), very similar to the 15.8% (2^0.212 ^= 1.158) higher concentration in cases compared to controls from the IPAS analyses of Table 1. Further analysis of case versus control log-ratios, which included the matching variables and several other CHD risk factors to control for possible confounding, produced similar findings (geometric mean of 1.275 with 95% CI of 1.122 to 1.450).

Based on individual plasma samples from 68 stroke cases occurring during the first year following randomization in the hormone therapy trials, and from 1-1 matched controls, ELISA evaluation yielded IGFBP4 concentrations that were 16.6% higher (*P *= 0.005) in cases versus controls (geometric mean of log-ratios of 1.166 with 95% CI of 1.050 to 1.295). The ELISA case versus control ratio was little altered by additional control for several other potential stroke confounding factors (geometric mean of 1.149 with 95% CI of 1.008 to 1.309 following this control).

**Figure 3:**
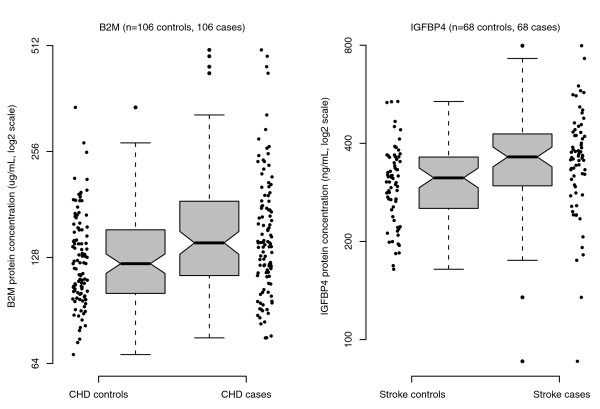
**Baseline plasma B2M concentrations for CHD cases and controls, and IGFBP4 concentrations for stroke cases and controls, from the Women's Health Initiative hormone therapy trials**. Individual ELISA-based concentrations are shown along with boxplots showing the median (dark line) and the 25th and 75th percentiles (bottom and top of box). The notches indicate 95% confidence intervals for the median.

Figure 3 shows the B2M assessments for individual CHD cases and controls and the IGFBP4 assessments for individual stroke cases and controls in these replication studies.

## Discussion

The proteomic discovery and replication studies presented here show plasma B2M to be a risk marker for CHD in postmenopausal women. B2M is an amyloidogenic protein that is elevated in hemodialysis patients and in patients having bone disease [26,27]. B2M has been reported to be associated with CHD risk factors, and an inverse association with HDL cholesterol [28]. Positive associations with peripheral arterial disease [29] and with total mortality among elderly Japanese men and women [30] have also been reported.

Our finding of B2M elevation in plasma obtained months or years prior to CHD diagnosis appears to be novel. Logistic regression analysis of ELISA B2M data yield odds ratios (95% CI) for the second, third, and fourth quartile of B2M, compared to the first, of 1.28 (0.46, 3.53), 1.77 (0.63, 4.96), and 3.40 (1.23, 9.35), with a trend test having *P *= 0.002, in analyses that control for case-control matching factors as well as hormone therapy randomization assignment, hysterectomy status, ethnicity, and history of myocardial infarction. From Table 3 we see that B2M levels increased by an estimated 15.5% (2^0.208 ^= 1.155) following E+P use and by 17.3% (2^0.230 ^= 1.173) following E-alone use. A 16% elevation in B2M projects a CHD odds ratio (95% CI) of 1.30 (1.11, 1.54) based on a logistic regression analysis with a linear term in log B2M, as determined by ELISA, and these same confounding control variables. Hence, the B2M elevation resulting from hormone therapy use could contribute importantly to an explanation for observed early elevations in CHD risk. The fact that CHD elevations evidently dissipate with longer-term hormone therapy use [5,6] could, for example, reflect concurrent favorable changes in plasma cholesterol fractions, especially for E-alone.

Our proteomic discovery work also suggests (Table 4; *P *= 0.03) higher B2M levels in stroke cases versus controls, so that this marker may help to understand adverse effects of hormone therapy on cardiovascular disease more generally. The B2M we identified in prediagnostic plasma samples likely differs from modified forms in non-osteotendinous fibrils or insoluble cardiac deposits [31]. However, B2M may provide a valuable focus for studies of disease mechanism and therapeutic intervention in spite of uncertainties about the relationship of plasma levels and pathophysiologic effects within tissue.

The discovery and replication studies presented here also show IGFBP4 to be a risk marker for stroke in postmenopausal women, which appears to be a novel finding. Logistic regression analyses that include a linear term in log IGFBP4 along with the case-control matching variables, hormone therapy randomization assignment, systolic and diastolic blood pressure, body mass index, and indicator variables for cigarette smoking, diabetes, and prior hormone therapy use yield a *P*-value of 0.018 for an association of IGFBP4 with stroke risk. A 20% increase in IGFBP4, as is consistent with the effects of E-alone and E+P on IGFBP4, projects an odds ratio (95% CI) of 1.40 (1.06, 1.85) in these analyses, suggesting that this marker could contribute importantly to a mechanistic explanation for the approximate 40% higher incidence of stroke among E-alone and E+P users in the WHI randomized trial [3,4]. Also, it is interesting that four of the eleven top-ranked proteins for association with stroke risk (Table 2) are members of the IGF signaling pathway (IGFBP4, IGF2, IGFBP6, IGFBP2). There have been some previous reports of associations between IGF pathway proteins and stroke [32-34]. Increased IGF binding protein levels may result in decreased IGF protein concentrations. IGF1 has been proposed as a potential neuroprotective protein for stroke [35].

To more directly assess the role of B2M and IGFBP4 in mediating hormone therapy effects on CHD and stroke, respectively, we are currently carrying out ELISA analyses of baseline and 1-year plasma samples in the WHI hormone therapy trials. The effect of changes between baseline and 1-year on these proteins on subsequent hormone therapy hazard ratios for CHD and stroke will be examined.

Other proteins having small FDRs for association with CHD (Table 1) or stroke (Table 2) will benefit from evaluation in replication studies. Some of these have previously received some consideration as vascular disease risk markers, including ORM1 [36-40], APOA2 [41-43], PPIA [44], and IGFALS [45-47].

In addition to protein set analyses based on KEGG pathways (described in Results), we also examined Gene Ontology [48] pathways related to inflammation. There was some evidence (*P *= 0.03) for a difference between CHD cases and controls for a cytokine activity pathway (CCL5, C5, PF4, and CCL16), and some (*P *= 0.04) for an acute inflammatory response pathway (ORM1, ORM2, C2, CFHR1, MBL2, AHSG), whereas there was no evidence of corresponding differences between stroke cases and controls.

## Conclusions

We have identified B2M and IGFBP4 as novel risk markers for CHD and stroke, respectively. These markers have potential to help elucidate hormone therapy effects on these diseases as observed in the WHI randomized controlled trials. The IPAS platform [11-14] provides quantification only for proteins having cysteine residues, but otherwise our analyses benefit from the depth of the proteomic profiling. Concentration ratios associated with hormone therapy in our earlier IPAS studies agreed closely with ELISA-based ratios from the same samples [9], and IPAS concentration ratios for E-alone and E+P agreed closely with each other for many proteins identified as hormone-therapy related. These comparisons suggest that a number of additional proteins with small FDRs (for example, < 0.2) in Tables 1 and 2 are likely also to be disease risk markers, though it will be important for these associations to be replicated in independent samples.

## Abbreviations

APOA2: apolipoprotein A-II precursor; B2M: beta-2 microglobulin; CHD: coronary heart disease; CI: confidence interval; E-alone: estrogen-alone; E+P: estrogen plus progestin; ELISA: enzyme-linked immunosorbent assay; FDR: false discovery rate; IGFALS: insulin-like growth factor-binding protein acid labile subunit; IGFBP4: insulin-like growth factor binding protein 4; IPAS: intact protein analysis system; IPI: International Protein Index; KEGG: Kyoto Encyclopedia of Genes and Genomes; MS/MS: tandem mass spectrometry; ORM1: alpha-1-acid glycoprotein 1; PPIA: peptidyl-prolyl isomerase A; WHI: Women's Health Initiative.

## Competing interests

The authors declare that they have no competing interests.

## Authors' contributions

RLP, LMA, LC, SJP (FHCRC), JH, RDJ, JER, JEM, CE, and SMH participated in drafting the manuscript. Data were collected, analyzed, and interpreted by RLP, SJP (University of Michigan), LMA, SJP (FHCRC), MM, TBB, KJ, and SMH. RLP and SMH were responsible for study design. Statistical analysis was performed by AA, LC, MM, PW, and RLP.

## Supplementary Material

Additional file 1 **Supplementary methods**. Detailed methods for sample preparation, protein fractionation, and mass spectrometry analysis are described. Click here for file

Additional file 2 **Table S1**. Baseline characteristics for women developing coronary heart disease (CHD) or stroke and for corresponding disease-free controls, drawn from the Women's Health Initiative Observational Study. Click here for file

Additional file 3 **Table S2**. CHD case versus control log-transformed concentration ratios for all quantified proteins. Click here for file

Additional file 4 **Table S3**. Stroke case versus control log(base2)-transformed concentration ratios for all quantified proteins. Click here for file
