# Supplementary material for: Novel proteins associated with risk for coronary heart disease or stroke among postmenopausal women identified by in-depth plasma proteome profiling
Source: Genome Med. 2010 Jul 28;2(7):48. doi: 10.1186/gm169 (PMC2923740; doi:10.1186/gm169)
Supplement: Additional file 1 — Supplementary methods. Detailed methods for sample preparation, protein fractionation, and mass spectrometry analysis are described. [file gm169-S1.DOC]

**SUPPLEMENTARY METHODS**

Sample Preparation

For each IPAS experiment 5 μL aliquots of EDTA plasma for each of the 100 cases or 100 controls were pooled. Case and control pools were separately immunodepleted to remove the top six most abundant proteins (albumin, IgG, IgA, transferring, haptoglobin, and antitrypsin) using a Hu-6 column (4.6 x 250 mm; Agilent). The immunodepleted samples were concentrated using Centricon YM-3 devices (Millipore) and re-diluted in 8 M urea, 30 mM Tris pH 8.5, 0.5% octyl-beta-d-glucopyranoside; Roche Diagnositcs). Samples were then reduced with dithiothreitol (DTT) using 0.66 mg DTT/mg protein. Isotopic labeling of cysteine residues of intact proteins was then performed with acrylamide. For CHD experiments, control pools received the light acrylamide isotope (C12 acrylamide; >99.5% purity; Sigma-Aldrich (Fluka), St. Louis, MO, USA), and case pools received the heavy 1,2,3-C13-acrylamide isotope (C13 acrylamide; >98% purity; Cambridge Isotope Laboratories). For the stroke experiments, the heavy and light acrylamide isotope were reversed for half the pools, so the control received the light isotope for 4 experiments, and the heavy isotope for the other 4 experiments. The pools of case and control samples were then mixed together in each experiment for further analysis.

Protein Fractionation

For each experiment, samples were fractionated in two dimensions, anion exchange chromatography, followed by reversed phase chromatography. For each IPAS experiment, the samples were diluted to 10 mL with 20 mM Tris in 6% isopropanol, 4 M urea, pH 8.5, and immediately injected onto a Mono-Q 10/100 column (Amersham Biosciences) for anion-exchange chromatography. 8 anion exchange fractions were collected and then subjected to a second dimension of separation by reversed-phase chromatography using a Poros R2 column (4.6x50 mm; Applied Biosystems) using trifluoroacetic acid/acetonitrile as the buffer system. Reversed phase fractions digested with trypsin (Promega) and pooled into 12 pools for each anion exchange fraction, resulting in a total of 96 fractions to be analyzed for each IPAS experiment.

Mass Spectrometry Analysis

Samples were analyzed using a linear ion trap – Fourier Transform (LTQ-FT) mass spectrometer (ThermoElectron) coupled to a 2D nanoLC system (Eksigent). Liquid chromatography separation was performed using a 25 cm column (Picofrit 75 μm internal diameter, New Objectives, packed in-house with Magic C18 resin). Spectra were acquired in data-depenent mode (m/z 400-1800) with sampling of the five most abundant doubly or triply charged ions of the MS1 for MS/MS analysis.
