# Supplementary material for: Novel proteins associated with risk for coronary heart disease or stroke among postmenopausal women identified by in-depth plasma proteome profiling
Source: Genome Med. 2010 Jul 28;2(7):48. doi: 10.1186/gm169 (PMC2923740; doi:10.1186/gm169)
Supplement: Supplementary file 2 [file gm169-S2.DOC]

Supplementary Table 1. Baseline characteristics for women developing coronary heart disease (CHD) or stroke and for corresponding disease-free controls, drawn from the Women's Health Initiative Observational Study

	CHD					Stroke				
	Case		Control		*P*-value	Case		Control		*P*-value
	N	%	N	%		N	%	N	%	
Age at baseline					1.00					1.00
50-59	96	12.0	95	11.9		81	10.1	82	10.3	
60-69	339	42.4	340	42.5		308	38.5	307	38.4	
70-79	365	45.6	365	45.6		411	51.4	411	51.4	
Race/ethnicity					1.00					1.00
White	674	84.3	674	84.3		656	82.0	656	82.0	
Black	75	9.4	75	9.4		90	11.3	90	11.3	
Hispanic	14	1.8	14	1.8		14	1.8	14	1.8	
Other	37	4.6	37	4.6		40	5.0	40	5.0	
Body-mass index (kg/m2)					0.14					0.68
< 25	270	34.2	287	36.5		290	37.0	299	38.0	
25 - < 30	258	32.7	275	35.0		267	34.1	275	35.0	
≥ 30	261	33.1	224	28.5		227	29.0	212	27.0	
Education					0.02					0.12
≤ High school/GED or less	240	30.2	196	24.8		175	22.1	208	26.2	
School after high school	299	37.6	297	37.6		311	39.3	282	35.6	
College degree or higher	256	32.2	297	37.6		305	38.6	303	38.2	
Smoking status					< .0001					0.15
Never	331	42.0	424	53.7		377	48.1	409	51.6	
Past	379	48.1	313	39.6		346	44.2	340	42.9	
Current	78	9.9	53	6.7		60	7.7	44	5.5	
Myocardial infarction ever	128	16.0	128	16.0	1.00	113	14.2	34	4.3	<0.0001
Stroke ever	42	5.3	17	2.1	0.001	117	14.6	117	14.6	1.00
Postmenopausal estrogen use					0.23					0.61
Never used	501	62.8	478	59.8		469	58.7	489	61.1	
Past user	117	14.7	112	14.0		133	16.6	125	15.6	
Current user	180	22.6	210	26.3		197	24.7	186	23.3	
Postmenopausal estrogen + progesterone use					0.33					0.30
Never used	660	82.5	644	80.5		671	84.0	652	81.5	
Past user	55	6.9	52	6.5		53	6.6	54	6.8	
Current user	85	10.6	104	13.0		75	9.4	94	11.8	

P-value based on chi-squared test of association.
